# Dreaming in Bipolar Disorders – Feasibility of the Central Image Method: A Prospective Controlled Trial

**DOI:** 10.3389/fpsyg.2024.1339734

**Published:** 2024-09-02

**Authors:** Gabriele Schmid-Mühlbauer, Laura Reggiannini, Peter Treu, Woo Ri Chae, Thomas Stamm

**Affiliations:** ^1^Klinikum Schloss Lütgenhof, Dassow, Germany; ^2^Charité – Universitätsmedizin Berlin, Department of Psychiatry and Psychotherapy, Berlin, Germany; ^3^International Psychoanalytic University Berlin, Berlin, Germany; ^4^Charité – Universitätsmedizin Berlin, Department of Psychiatry and Neurosciences, Berlin, Germany; ^5^DZPG, German Center for Mental Health, Berlin, Germany; ^6^Berlin Institute of Health at Charité – Universitätsmedizin Berlin, BIH Biomedical Innovation Academy, BIH Charité Clinician Scientist Program, Berlin, Germany; ^7^Medizinische Hochschule Brandenburg Theodor Fontane, Neuruppin, Germany

**Keywords:** emotion, dream, bipolar disorder, prospective controlled trial, Central Image Method

## Abstract

**Background:**

Emotions play an important role in the emergence, formation, and experience of dreams. The Central Image (CI) in a dream refers to the dream’s dominant or underlying emotion, and it is the best-remembered part of a dream. Bipolar disorders are characterized by strong emotions, particularly during manic and depressive episodes. In these patients, dreams and CIs may serve as a helpful diagnostic and therapeutic tool. This study aims to evaluate the feasibility of the CI Method and to check for differences concerning dreams and CI emotions between healthy controls and patients with bipolar disorder.

**Methods:**

Over a period of 3 weeks, 61 participants were asked to maintain a daily record of all dreams remembered. Dream reports were rated using the Central Image Method (CIM) by two independent, blinded raters. We assessed for interrater agreement on the CIM and for within and between group differences related to negative and positive CI emotions.

**Results:**

Mean agreement rates (weighted Cohen’s kappa) for the CI emotion ratings could be classified as very good: For primary CI emotion ratings, the mean weighted Cohen’s kappa was 0.99 (± 0.02), and for secondary CI emotions, it was 0.90 (± 0.17). Regarding the CI intensities, the mean agreement rate (Spearman’s correlation) was also strong. Evaluation of differences within the groups showed that there were more negative CI emotions than positive CI emotions in healthy controls and currently depressive patients; however, in the latter, the difference was statistically not significant. Analyses of differences between groups indicated that patients who were currently depressed recorded more negative CI emotions in dreams than those who were euthymic or healthy controls.

**Discussion:**

The findings support the feasibility of the CIM. Our results might indicate different emotion regulation and defense mechanisms across bipolar disorder states, as reflected by the occurrence of negative and positive CI emotions.

## Introduction

Dreams may be defined as a (recallable) mental activity that occurs during sleep (Wittmann et al., 2007; Schredl, 2009; Euler et al., 2016). Emotions play an important role in the emergence and formation of dreams, and at the same time, they are part of the dream experience (Reiser, 2001), particularly during rapid eye movement (REM) sleep dreaming (Sikka et al., 2019). Cross-cultural and cross-gender similarities have been found in dreams. For example, there are usually more negative than positive emotions in our dreams (Beauchemin and Hays, 1995; Domhoff, 2001).

While Freud is considered to have pioneered dream research (Freud, 1991), since then a range of dream theories have evolved (Deserno, 1999; Mertens, 2009). Current dream research focuses on the role of dreams in terms of information processing, memory consolidation, and emotion/mood regulation. There is preliminary evidence that dreaming (beyond sleep) might be useful for emotion regulation processes through a reorganization of experience, affective stimulation, or recalibration (Sterpenich et al., 2020; Mariani et al., 2021). In addition, dreaming might have a “quasi-therapeutic function,” e.g., concerning the integration of traumatic events into experiences of one’s own life (Hartmann, 1998). Eichenlaub et al. (2018) found an association between frontal theta activity and recent waking-life experiences in REM dreams but no link for older memories, indicating a transformation of memories in REM sleep. The authors concluded that dreaming during REM sleep might reflect emotional memory processing (Eichenlaub et al., 2018).

Hartmann postulated that dreams have a restorative and adaptive function by increasing connections, weaving in something new, and broadening memory (Hartmann, 1996). He presumed an auto-therapeutic function of dreams, i.e., dreams are helpful in processing threatening or irritating experiences and in connecting these with other, less distressing memories (Roesler, 2023). Experienced emotions and dreams are highly connected: “*Dreams contextualize (find a picture context for) the emotional concern*” (Hartmann, 1998), e.g., a tidal wave in a dream might picture a person’s emotional state of being or feeling overwhelmed. The “*Central Image*” (CI) or contextualizing image is “*a striking, arresting, or compelling image—not simply a story—but an image that stands out by virtue of being especially powerful, vivid, bizarre, or detailed*” (Hartmann and Brezler, 2008). The CI is the dominant or underlying emotion of the dream and is the best-remembered part of the dream (Hartmann and Kunzendorf, 2006). It might be considered that the intensity of the CI is associated with the emotional activation or strength of the underlying emotion (Hartmann et al., 2001; Hartmann and Brezler, 2008).

In the *continuity hypothesis,* it has been proposed that waking-life experiences, such as concerns, thoughts, actions, and emotions, are reflected in dreams (Hall and Nordby, 1972; Hartmann, 2011; Schredl, 2012). Thus, in the case of mental health difficulties, an individual’s psychopathological symptoms should also be reflected in an individual’s dreams and dream reports. In post-traumatic stress disorder (PTSD), post-traumatic nightmares, i.e., frightening dreams with awakening, are common (Wittmann et al., 2007). Akkaoui et al. conducted a systematic review and found that nightmares were overrepresented in mood and psychotic disorders, whereby distress associated with nightmares was linked with the severity of the disorder (Akkaoui et al., 2020). Depressive disorders are often related to negatively altered dreams, and antidepressants may reduce REM sleep and decrease dream recall (Tribl et al., 2013). One study with psychiatric inpatients and healthy controls revealed that dreams of depressed patients were more likely to comprise themes of depression. Additionally, the severity of specific symptoms seemed to be associated with the dream content (Schredl and Engelhardt, 2001). It was found that individuals with schizophrenia reported more aggressive social interactions and more negative emotions in their dreams compared to depressed people (Kramer and Roth, 1973). In a preliminary study, the dream themes and mood states of six outpatients with bipolar disorder were evaluated, finding that manic states were associated with bizarre and unlikely dream contents. Additionally, dreams of death and bodily injury preceded mood shifts, namely to mania, while a decreased overall number of dreams reported was an early symptom of depressed mood (Beauchemin and Hays, 1995).

Bipolar disorders are severe chronic mood disorders characterized by episodes of (hypo-)mania, episodes of depression, and mixed states. According to the current Diagnostic and Statistical Manual of Mental Disorders (DSM-5), in bipolar I disorder, at least one manic episode must have occurred, and while major depressive episodes are typical, they are not required to meet a diagnosis. In bipolar II disorder, at least one hypomanic episode and one major depressive episode must have occurred (Grande et al., 2016). Generally, patients with bipolar disorders experience episodes of extreme emotions and fluctuations in their emotions.

The purpose of the prospective controlled study on Dreams and Bipolar Disorders (TBS study, German: ***T**räume und **B**ipolare **S**törung*) is to investigate whether there are associations between conscious and unconscious emotions in different states of bipolar disorders compared to healthy controls.

The first aim of this paper is to evaluate the feasibility of the Central Image Method (CIM) in terms of dreaming in healthy controls and individuals with bipolar disorders during states of euthymia, depression, and (hypo)mania. We want to examine:

(a) whether these groups differ in terms of the number of dreams experienced,
(b) whether these groups differ in the number of negative and positive CI emotions experienced, and
(c) whether currently (hypo-)manic individuals would experience more positive CI emotions in their dreams as compared to the other three groups.

## Materials and methods

### Sample and study design

This prospective controlled trial was conducted between August 2013 and September 2015 in Berlin, Germany. Patients were mainly recruited via the specialized outpatient service for bipolar disorders at the Charité University Hospital’s Department of Psychiatry and Psychotherapy and underwent psychiatric examination.

Healthy controls were recruited through private contacts in Berlin, announcements at hospitals and universities in Berlin, and student mailing lists from the Charité University Medicine, Berlin, and the International Psychoanalytic University in Berlin.

All participants were informed about the study and its aims and had to provide their written informed consent to participate in the study. The TBS study was reviewed and approved by the ethics committee of the Charité University Medicine, Berlin (Reference number: EA4/068/13).

To address our research aims, we divided the sample into four groups: (1) healthy control, (2) bipolar disorder, currently euthymic, (3) bipolar disorder, currently depressive, and (4) bipolar disorder, currently (hypo-)manic.

### Inclusion and exclusion criteria

The inclusion and exclusion criteria for the healthy control group and the patient groups are shown in Table 1.

**Table 1:** Inclusion and exclusion criteria.

	Patient groups	Healthy control group
Inclusion criteria	Age of ≥18 years	Age of ≥18 years
	Diagnosis of bipolar affective disorder according to ICD-10 (World Health Organisation, 2012). **Subgroups were classified as follows: Currently** **euthymic** for at least 3 months; currently no affective symptoms; pharmacotherapy with at least one mood stabilizer for at least 3 months; last dose change at least 14 days ago **depressive** episode: HAMD-21 ≥ 15 (Hamilton, 1960) **(hypo-)manic** episode: YMRS ≥12 (Young et al., 1978)	Lifetime absence of psychiatric disorders as assessed by a short diagnostic interview for mental disorders (Mini-DIPS) (Margraf, 1994) and the SCID-II (screening for personality disorders corresponding to the DSM-IV) (Wittchen et al., 1997).
Exclusion criteria	Current mixed episode (HAMD-21 ≥ 10 and YMRS ≥12)	Lifetime psychiatric diagnosis as assessed by Mini-DIPS (Margraf, 1994) **or** the SCID-II (Wittchen et al., 1997).
	Current psychotic symptoms	
	Current substance abuse (aside from caffeine and nicotine)	
	Within the last 6 months, the prevalence of another axis I disorder	
	Diagnosis of an antisocial personality disorder according to ICD-10	
	Diagnosis of dementia or a mild cognitive disorder according to ICD-10	
	Electroconvulsive therapy within the last 6 months	

HAMD-21, Hamilton Depression Rating Scale, 21-item version; YMRS, Young Mania Rating Scale; Mini-DIPS, Short diagnostic interview for mental disorders; SCID-II, SCID-II – Structured Clinical Interview for DSM-IV, part II.

### Instruments

The instruments included in this study are the following standardized interviews and questionnaires, completed at baseline unless otherwise stated.

#### All participants

The *Basic Documentation (BaDo)* case report form was used to assess sociodemographic data, e.g., age, gender, and employment status.

The *NEO Five-Factor Inventory (NEO-FFI)* is a widely used, self-rating questionnaire for assessing personality characteristics (Borkenau and Ostendorf, 2007). The following five dimensions are measured: neuroticism, extraversion, openness to experience, agreeableness, and conscientiousness.

The *Positive and Negative Affect Schedule (PANAS)* is a 20-item, self-rating questionnaire for assessing positive and negative emotional states. Ten of these refer to positive states (e.g., interested, strong) and 10 refer to negative states (e.g., distressed, afraid), each rated on a five-point scale (1 = not at all or very slightly; 2 = a little; 3 = moderately; 4 = quite a bit; 5 = extremely) (Watson et al., 1988; Krohne et al., 1996). The PANAS was recorded on 3 of the 21 days (chosen at random). The PANAS was used to validate the self-rating questionnaire *Emotions of the day,* which was particularly designed for the TBS study; it is described in the following section.

*Emotions of the day*. This novelly designed questionnaire taps into 18 emotions according to the CIM. Every evening before going to sleep for the 21 days of the study, participants were asked to rate the following 18 emotions on a five-point scale (1 = very slightly or not at all; 2 = a little; 3 = moderately; 4 = quite a bit; 5 = extremely). Second, the three strongest emotions of the day should be marked and ranked (i.e., 3 = strongest emotion, 2 = second strongest, 1 = third strongest).

*Dream diary*. For a period of 3 weeks, all participants had to keep a handwritten record of their dream(s) immediately after waking up in the morning. In addition, they should rate their dream emotions using the *Emotions while dreaming* sheet, which was novelly designed for the TBS study. It is described in the following section.

*Emotions while dreaming (i.e., intensity ratings of dreams and dream emotions)*. A self-rating questionnaire was designed for the TBS study based on the emotion theory of Ekman (1992) to assess the intensity of dreams and dream emotions. Daily, the participants should rate the intensity of the dream of the previous night on a 10-point scale (0 = not at all intense to 10 = strongly intense) and the intensity of the six emotions, fear, anger, joy, sadness, disgust, and surprise while dreaming, on a 10-point scale (0 = not at all intense to 10 = strongly intense).

#### Healthy controls

*Short Diagnostic Interview for Mental Disorders (Mini-DIPS, German: Diagnostisches Kurz-Interview bei Psychischen Störungen)* is a short diagnostic interview according to the diagnostic criteria of DSM-IV and ICD-10 (Margraf, 1994).

The *Structured Clinical Interview for DSM-IV – part II (SCID-II)* aims to screen for and assess personality disorders (Wittchen et al., 1997). The two-step procedure consists of a questionnaire and a subsequent standardized interview.

#### Patients

The *Hamilton Depression Rating Scale, 21-item version* (*HAMD-21*), is an external rating instrument to assess the severity of depressive symptoms in the last 7 days (Hamilton, 1960). The 21 items are summed up; a sum score from 0 to 9 means no depression, 10 to 19 mild, 20 to 29 moderate, and > 29 severe symptoms of depression. The HAMD-21 was used to monitor patients’ illness at three measurement points during the study.

The *Young Mania Rating Scale (YMRS)* is an external rating instrument for assessing the severity of manic symptoms in the last 7 days (Young et al., 1978). It consists of 11 items, which are summed up. A sum score of ≥12 indicates clinically relevant hypomanic symptoms, while ≥20 indicates manic pathology.

### Central Image Method (CIM)

The CIM aims to evaluate and quantify dream images in terms of intensity and underlying primary emotions.

*Procedure within the TBS study:* Two independent and blinded raters (co-authors of this study—L.R. and W.R.C.), who neither knew the dreamers/participants nor their emotional states, assessed whether the dream reports included a central image and, if so, identified the CI. In addition, they rated the emotion that might be expressed by the CI using a list with the following 18 emotions (Hartmann and Brezler, 2008): (1) fear, terror; (2) helplessness, vulnerability or being trapped, or immobilized; (3) anxiety, vigilance; (4) guilt; (5) grief, loss, sadness, abandonment, disappointment; (6) despair, hopelessness (giving up); (7) anger, frustration; (8) disturbing—cognitive dissonance, disorientation, weirdness; (9) shame, inadequacy; (10) disgust, repulsion; (11) power, mastery, supremacy; (12) awe, wonder, mystery; (13) happiness, joy, excitement; (14) hope; (15) peace, restfulness; (16) longing; (17) relief, safety; and (18) love (relationship), and in addition, 0) No CI. Moreover, they could name and rate a second emotion if another strong emotion was contextualized within the CI. Finally, they estimated the intensity of the CI on a seven-point scale ranging from 0 (no CI) to 3 (very strong CI intensity). In sum, the two raters independently checked all dream reports in terms of whether a CI occurred, and, if a CI occurred on a certain day, they also rated the one or two main CI emotion(s) and the CI intensity.

### TBS study procedure

**Figure 1:**
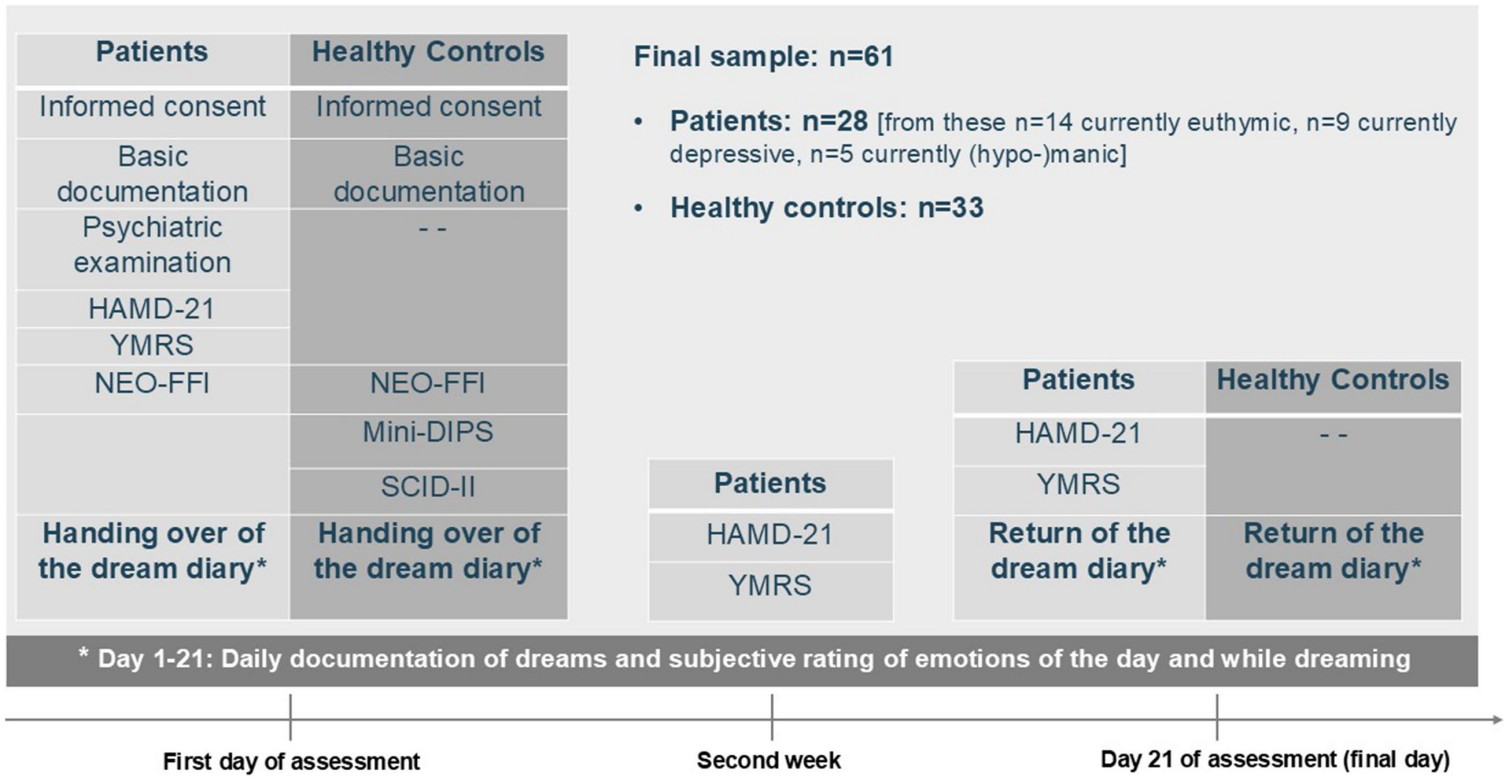
Study description–design, instruments, and assessment points. HAMD-21, Hamilton Depression Rating Scale, 21-item version; YMRS, Young Mania Rating Scale; NEO-FFI, NEO Five-Factor Inventory; Mini-DIPS, Short diagnostic interview for mental disorders; SCID-II, Structured Clinical Interview for DSM-IV, part II.

The procedure and the measurements/instruments slightly differed between the control and the patient group. Figure 1 shows the timeline and study contents for the controls and patients.

### Analyses

All statistical analyses were performed using IBM SPSS Version 27. Descriptive statistics concerning sociodemographic data and characteristics of the patient subgroups (i.e., type of bipolar disorder, current medication, and HAMD-21 and YMRS sum scores) are presented in Tables 2, 3. In addition, differences between groups were evaluated, namely between (1) healthy control and patient groups and (2) patient group and dropouts/excluded using the chi-square tests and non-parametric Mann–Whitney U-tests (see Table 2). Differences between patient groups concerning medication proportions were analyzed using the chi-square tests (see Appendix).

**Table 2:** Sample description and differences between groups.

	Healthy control group	Patient group	Dropouts/excluded^a^	Differences between the healthy control group and the patient group	Differences between the patient group and dropouts/excluded
	*n* = 33	*n* = 28	*n* = 12		
Age (years) M ± SD	25.5 ± 7.7	46.4 ± 14.6	39.7 ± 14.0	*U* = 63.0; *Z* = −5.8; ***p* < 0.001**	*U* = 124.0; *Z* = −1.3; *p* = 0.19
(range)	(18; 60)	(25; 73)	(23; 60)		
Gender n (%)					
Male	9 (27.3%)	9 (32.1%)	7 (58.3%)	Χ^2^ = 0.34; df = 1; *p* = 0.56	Χ^2^ = 7.9; df = 1; ***p* = 0.005**
Female	24 (72.7%)	19 (67.9%)	5 (41.7%)		
Highest school degree n (%)					
Middle school or secondary School diploma	2 (6.1%)	5 (17.9%)	4 (33.3%)	Χ^2^ = 6.8; df = 1; ***p* = 0.009**	Χ^2^ = 3.0; df = 1; *p* = 0.08
High school/technical diploma	31 (93.9%)	23 (82.1%)	8 (66.7%)		
Type of episode n (%)					
Euthymic	–	14 (50.0%)	6 (50.0%)	–	Χ^2^ = 6.0; df = 2; ***p* = 0.049**
Depressive	–	9 (32.1%)	2 (16.7%)		
(Hypo-)manic	–	5 (17.9%)	4 (33.3%)		

^aDropout/excluded only from the patient group. Significant p-levels are in bold.^

**Table 3:** Patients’ clinical characteristics and medication use.

	Total	Euthymic	Depressive	(Hypo-)manic
	*n* = 28	*n* = 14	*n* = 9	*n* = 5
Bipolar disorder				
Type I	16	7	6	3
Type II	12	7	3	2
Medication^a^ n (%)				
Lithium	10 (35.7%)	8 (57.1%)	1 (11.1%)	1 (20.0%)
Antiepileptics	16 (57.1%)^b^	4 (28.6%)	7 (77.8%)^b^	5 (100.0%)
Antipsychotics/neuroleptics	17 (60.7%)^c^	7 (50.0%)^c^	6 (66.7%)	4 (80.0%)
Low-potency neuroleptics	0 (0.0%)	0 (0.0%)	0 (0.0%)	0 (0.0%)
Antidepressants	15 (53.6%)^d^	7 (50.0%)^d^	7 (77.8%)^d^	1 (20.0%)
Benzodiazepines, Z-drugs	2 (7.1%)	0 (0.0%)	0 (0.0%)	2 (40.0%)
HAMD-21 sum score M ± SD (range), n				
1st assessment point	9.8 ± 10.5 (0; 35), *n* = 27	3.4 ± 3.1 (0; 9), *n* = 14	23.1 ± 6.2 (13; 35), *n* = 9	2.3 ± 3.3 (0; 7), *n* = 4
2nd assessment point	7.7 ± 8.8 (0; 30), *n* = 25	2.0 ± 2.2 (0; 7), *n* = 13	18.1 ± 6.0 (10; 30), *n* = 9	1.3 ± 1.2 (0; 2), *n* = 3
3rd assessment point	7.6 ± 8.3 (0; 23), *n* = 23	2.6 ± 3.9 (0; 10), *n* = 13	17.1 ± 5.2 (10; 23), *n* = 8	1.5 ± 2.1 (0; 3), *n* = 2
YMRS sum score M ± SD (range), n				
1st assessment point	4.1 ± 7.9 (0; 29), *n* = 27	0.8 ± 2.0 (0; 7), *n* = 14	1.9 ± 4.0 (0; 11), *n* = 9	20.5 ± 7.3 (13; 29), *n* = 4
2nd assessment point	2.8 ± 5.9 (0; 24), *n* = 25	0.7 ± 1.8 (0; 6), *n* = 13	1.1 ± 2.7 (0; 8), *n* = 9	16.7 ± 6.7 (11; 24), *n* = 3
3rd assessment point	2.2 ± 5.2 (0; 19), *n* = 23	1.5 ± 4.1 (0; 15), *n* = 13	0.3 ± 0.7 (0; 2), *n* = 8	15.0 ± 5.7 (11; 19), *n* = 2

^a^Multiple answers were possible; in addition, some patients took more than one drug of one substance class. ^b^One patient from the depressive group with two antiepileptics (valproate and lamotrigine). ^c^One patient from the euthymic group with two antipsychotics (aripiprazole and clozapine). ^d^Three patients with two antidepressants, namely one from the euthymic group (agomelatine and mirtazapine) and two from the depressive group (both with escitalopram and bupropion). HAMD-21, Hamilton Depression Rating Scale; 21-item version; YMRS, Young Mania Rating Scale.

#### Feasibility of CIM

Number of dreams and rating of occurrence of CI. CI emotion(s) were judged by two independent and blinded raters. The interrater agreement was determined using the weighted Cohen’s kappa (Κ). Κ can be classified as follows: No/weak agreement if Κ ≤ 0.20; small if 0.21 ≤ Κ ≤ 0.40; moderate if 0.41 ≤ Κ ≤ 0.60; good if 0.61 ≤ Κ ≤ 0.80; or very good if Κ ≥ 0.81 (Grouven et al., 2007). Only if both CI emotion ratings matched, ratings were considered for further analyses. Furthermore, for CI intensities, the interrater agreement was evaluated using Spearman’s correlation coefficient ρ, which can be classified as follows: small correlation if |0.10| ≤ ρ < |0.30|; moderate correlation if |0.30| ≤ ρ < |0.50|; strong correlation if ρ ≥ |0.50|(Cohen, 1988).

#### Group differences in the number of dreams and CI emotions

Differences between the groups were evaluated using the non-parametric Kruskal–Wallis test (k independent groups) and, if needed, the Dunn–Bonferroni *post-hoc* tests (Bonferroni adjusted level of significance) (see Tables 4, 5).

**Table 4:** Number of dreams and differences between groups.

	Healthy control group	Euthymic	Depressive	(Hypo-)manic	Non-parametric Kruskal–Wallis Test	Dunn-Bonferroni *post-hoc* tests^b^	
	*n* = 33	*n* = 14	*n* = 9	*n* = 5			
No. of dreams	186	100	88	53			
M ± SD (range)	5.6 ± 3.8 (1; 17)	7.1 ± 6.2 (1; 19)	9.8 ± 4.7 (0; 16)^a^	10.6 ± 4.6 (5; 16)	Kruskal Wallis H = 8.2;df = 3; ***p* = 0.04**	C vs. E	*p* = 1.00
						C vs. D	*p* = 0.15
						C vs. (H)M	*p* = 0.21
						E vs. D	*p* = 0.67
						E vs. (H)M	*p* = 0.64
						D vs. (H)M	*p* = 1.00

^a^One patient from the depressive subgroup did not report any dreams. ^b^p is Bonferroni adjusted; C, Healthy Control group; E, Euthymic; D, Depressive; (H)M, (Hypo-)manic. Significant *p*-levels are in bold.

**Table 5:** Negative and positive CI emotions—differences between and within groups.

	Healthy Control group	Euthymic	Depressive	(Hypo-)manic	Non-parametric Kruskal–Wallis Test	Dunn–Bonferroni *post-hoc* tests^b^	
	*n* = 33	*n* = 14	*n* = 8^a^	*n* = 5			
Negativity index					Kruskal Wallis H = 3.2; df = 3; *p* = 0.37	–	–
M ± SD	0.75 ± 0.27	0.60 ± 0.38	0.77 ± 0.15	0.62 ± 0.06			
Positivity index					Kruskal Wallis H = 3.2; df = 3; *p* = 0.37	–	–
M ± SD	0.25 ± 0.27	0.40 ± 0.38	0.23 ± 0.15	0.38 ± 0.06			
No. of negative CI emotions	98	35	61	28			
M ± SD (range)	3.0 ± 1.9 (0; 8)	2.5 ± 2.2 (0; 8)	7.6 ± 3.5 (1; 13)	5.6 ± 1.9 (3; 8)	Kruskal Wallis H = 17.0; df = 3; ***p* = 0.001**	C vs. E	*p* = 1.00
Median	3.0	2.5	8.5	5.00		C vs. D	***p* = 0.01**
						C vs. (H)M	*p* = 0.21
						E vs. D	***p* = 0.01**
						E vs. (H)M	*p* = 0.08
						D vs. (H)M	*p* = 1.00
No. of positive CI emotions	43	34	16	18			
M ± SD (range)	1.3 ± 1.4 (0; 5)	2.4 ± 3.1 (0; 11)	2.0 ± 1.3 (0; 4)	3.6 ± 1.7 (2; 6)	Kruskal Wallis H = 7.6; df = 3; *p* = 0.06	–	–
Median	1.0	1.0	2.0	4.0			
Wilcoxon signed Rank test ^c^	z = −3.9; ***p* < 0.001**	z = −0.4; *p* = 0.69	z = −2.4; *p* = 0.02	z = −2.0; *p* = 0.04			

^a^One patient from the depressive subgroup did not report any dreams. ^b^p is Bonferroni adjusted. C, Healthy Control group; E, Euthymic; D, Depressive; (H)M, (Hypo-)manic. ^c^Evaluation of differences within groups concerning the number of negative vs. positive CI emotions; significance level p was set at 0.05/4 = 0.013. Significant *p*-levels are in bold.

For negative and positive CI emotions, the CI emotion ratings were dichotomized (modified from Hartmann et al., 2001): CI emotions 1-10 = negative CI emotions, and CI emotions 11-18 = positive CI emotions. In addition, negativity and positivity indices were computed, i.e., a quotient composed of the number of negative- or positive-rated CI emotions divided by the number of all CI emotions. Descriptive statistics were computed; differences between the subgroups were evaluated using the non-parametric Kruskal–Wallis test (k independent groups) and, if needed, the Dunn–Bonferroni *post-hoc* tests (Bonferroni-adjusted level of significance). Moreover, the non-parametric Wilcoxon signed-rank tests were conducted to check for within-group differences concerning the number of negative and positive CI emotions. Due to multiple testing, the significance level of p was set at 0.05/4 = 0.013 (see Table 5).

## Results

### Sample description

While 73 participants initially gave informed consent, 10 patients dropped out (resulting in 33 healthy controls and 30 patients). The reasons for dropout were symptom-specific (e.g., rapid cycling), deterioration of wellbeing due to focusing on dreams, chronic suicidality, or lack of motivation. In addition, two participants were excluded from the analyses due to a delay in filling out the dream diary (the first dream in September 2013 and the last dream in December 2013) and for not filling out any self-rating questionnaire.

There were significant demographic differences between the participating patient group (*n* = 28) and those patients who dropped out or were excluded (*n* = 12), namely concerning gender (with more male patients being excluded) and current type of episode (more (hypo-)manic individuals excluded) (see Table 2).

The final sample for analyses comprised 61 participants, including 28 bipolar patients and 33 healthy controls. Further sociodemographic information is available in Table 2, while Table 3 shows patients’ clinical characteristics and medication use. At measurement point three (final day of assessment), one patient from the euthymic group had a YMRS score of 15, indicating a hypomanic mood state.

Differences between patient groups concerning medication proportions are shown in Appendix.

All healthy controls fulfilled the inclusion criteria, i.e., ≥18 years of age and lifetime absence of psychiatric disorders as assessed by a short diagnostic interview for mental disorders (Mini-DIPS) (Margraf, 1994) and the SCID-II (screening for personality disorders corresponding to the DSM-IV) (Wittchen et al., 1997).

### Feasibility of CIM

In total, 427 dreams were reported. Of these, 186 dreams were recorded by the healthy control group, and 241 were recorded by bipolar patients (euthymic: 100, depressive: 88, hypo−/manic: 53). One depressed patient reported experiencing no dreams during the 21-day study period.

Three hundred and thirty-three primary and secondary CI emotions were identified by the raters. Of these, 141 were recorded by individuals from the healthy control group, while 192 were recorded by patients (further broken down as follows: euthymic: 69, depressive: 77, and hypo−/manic: 46).

Concerning the agreement of primary CI emotion ratings, the mean weighted Cohen’s kappa was 0.99 (± 0.02), ranging between 0.94 ≤ Κ ≤ 1.00; this can be classified as very good agreement. The mean interrater agreement regarding the secondary CI emotions was 0.90 (± 0.17), ranging from 0.38 to 1.00, which is a very good mean interrater agreement as well.

#### CI intensity

Regarding the CI intensities, the mean agreement rate (Spearman’s correlation) between the two independent, blinded raters was ρ = 0.93 (± 0.05), which can be classified as strong. The correlation coefficients ρ ranged from 0.85 to 1.00, indicating a high agreement rate.

### Dreams and CI emotions

#### Number of dreams

Table 4 shows the number of dreams in each group and the mean and standard deviations. The Kruskal–Wallis test indicated that the number of dreams differed significantly between the four groups, i.e., healthy controls and euthymic, depressive, and (hypo-)manic patients. However, the Dunn–Bonferroni *post-hoc* tests did not reveal any significant differences between the subgroups.

#### Negative/positive CI emotions—differences between groups

The 333 CI emotions (with interrater agreements) were divided into two categories: *negative CI emotions* and *positive CI emotions*. In the healthy control group, 98 negative CI emotions and 43 positive CI emotions were identified (totaling 141 CI emotions), and in the patient groups, 124 negative CI emotions [euthymic: 35; depressive: 61; (hypo-)manic: 28] and 68 positive CI emotions turned out [euthymic: 34; depressive: 16; (hypo-)manic: 18] (totaling 192 CI emotions) (see Table 5).

Regarding the average number of negative CI emotions, there were significant differences between the depressive and the healthy control group and between the depressive and the euthymic group: In depressive patients, on average, more negative CI emotions were found. Concerning the average number of positive CI emotions, there were no differences between the four groups (see Table 5).

#### Negative/positive CI emotions—differences within groups

The non-parametric Wilcoxon signed rank tests revealed that in the healthy control group, significantly more negative than positive CI emotions were identified. In the depressive group, more negative than positive CI emotions occurred; however, the difference was statistically not significant. The were no significant differences in the euthymic or (hypo-)manic groups concerning the number of negative and positive CI emotions (see Table 5).

## Discussion

The aim of the TBS study was to evaluate associations between conscious and unconscious emotions in patients with bipolar disorders as compared to healthy controls. To our knowledge, this is one of the rare studies dealing with dreams and emotions in different states of bipolar disorders.

Our principal findings were the following: The CIM proved to be feasible for examining dreaming in patients with bipolar disorders and healthy controls. The number of reported dreams differed between the groups; however, these differences were statistically insignificant. Evaluation of differences within subgroups revealed that there were more negative compared to positive CI emotions in healthy controls and depressive patients; however, in the latter, the difference was statistically not significant. Analyses of differences between groups indicated that there were more negative CI emotions in the dreams of depressive patients than in euthymic patients and healthy controls.

### Feasibility of CIM

Patients in different states of bipolar disorders, i.e., euthymic, depressive, and (hypo-)manic, as well as healthy controls, wrote down their dreams upon waking up for 21 consecutive days (with averages ranging from 5.6 dreams for healthy controls to 10.6 for (hypo-)manic patients). Only one individual (a depressive patient) did not remember or note down any dreams. This result can be seen as a sign of the acceptability of the method by the participants (Bowen et al., 2009).

The two raters were blinded concerning patients/controls and rated independently. The mean interrater agreements concerning CI emotions and CI intensities could be classified as very good or strong, indicating the high reliability and strength of the CIM (Hartmann et al., 2001). The high levels of interrater agreement indicate the potential for consistent and reliable application of the CIM.

### Dream recall in healthy controls and patients with bipolar disorder

The mean number of reported dreams was highest in (hypo-)manic patients, followed by depressive and, in third place, euthymic patients. Healthy controls, on average, reported the fewest dreams; however, *post-hoc* tests did not reveal any significant differences between the four groups. This result suggests that the ability to remember and recall dreams was not altered or reduced in subgroups of bipolar disorders compared to healthy controls. Some previous studies found reduced dream recall frequency in depressive patients (e.g., Armitage et al., 1995). Dream recall might be influenced by nocturnal awakenings, sleep quality, and personality (i.e., “thin” boundaries) (Schredl, 1999; Hartmann and Kunzendorf, 2006). When these variables were controlled, Schredl and Engelhardt (2001) did not find any differences regarding dream recall frequency between healthy controls and patients suffering from major depression or other mental disorders.

The higher ability to remember and recall dreams in the currently symptomatic subgroups [i.e., depressive and (hypo-)manic] might be a reflection of the auto-therapeutic function of dreams (Hartmann et al., 2001; Roesler, 2018, 2023). While dreaming, the brain does not have to process new input but can focus on problem-solving, i.e., irritating experiences are processed and new connections are made. This is guided by a central emotion, leading to an integration of threatening or irritating emotions. Dreams can be seen as creative and prospective narratives, i.e., trial runs in a safe place (Vedfelt, 2020). In their dreams, patients might experience problem-solving (Böker, from Deserno, 1999).

### Dreams and CI emotions in healthy controls and patients with bipolar disorders

In healthy controls, more negative than positive CI emotions were found. This result is according to previous studies (Beauchemin and Hays, 1995; Domhoff, 2001) and might be a sign of problem-solving and emotion regulation while dreaming (Mertens, 2009). In depressive patients, more negative than positive CI emotions were also detected, even though the difference was statistically not significant. Aside from the aspect of problem-solving and emotion regulation, this finding may support the continuity hypothesis postulating that waking-life experiences, symptoms, and emotions are replicated in dreams, and the severity of depressive symptoms might be associated with the intensity of negative emotions in the dream (Schredl and Engelhardt, 2001; Hartmann, 2011; Schredl, 2012).

Dreams may represent the state of an individual’s inner world (Roesler, 2023). In euthymic—symptom-free (also reflected by the initial psychometric results on the YMRS and HAMD-21, indicating “normal”/healthy functioning)—patients, we found almost balanced average numbers of positive and negative CI emotions. (Hypo-)manic patients also didn’t experience more negative CI emotions than positive ones. This result might indicate different emotion regulation or defense mechanisms in the subgroups of bipolar disorders, as reflected by the occurrence of negative and positive CI emotions. Mentzos (2002) postulated that psychoses, including manic-depressive psychoses, are mainly defense mechanisms that are actively mobilized against an intrapsychic tension; this tension results from inner psychic bipolarities or dilemmas, i.e., self-identity vs. attachment, that threaten the self and its integration. Psychiatric symptoms might be pictured in dreams, particularly if these symptoms are emotionally meaningful for the individual (Hartmann et al., 2001).

In bipolar disorders, the identification of early warning signs has been shown to be important in terms of relapse prevention (Morriss et al., 2007). Dreams, changes in dreams, and the (unconscious) emotions while dreaming might further play a role in diagnostic and therapeutic interventions for bipolar disorder and in supporting relapse prevention.

### Limitations and strengths

There are several limitations to our study. First, dreams were written down by participants. This approach is limited by the recall abilities of the participants and their writing skills. There might be differences between the actual dream and the dream report. In addition, motivational problems could also lead to bias. It is important to consider *how* the dream is reported: In prospective analyses or studies, the language should be analyzed in detail to try and account for reporting issues. Second, due to small patient groups, we (a) combined manic and hypomanic patients in one group and (b) were not able to control for confounders, e.g., age, gender, medication, or length of dreams. This should be considered in the interpretation of the results, particularly as there were significant differences concerning medication proportions between patient groups. Third, the healthy control group differed from the patient group concerning age and highest school degree. This should also be considered when interpreting the results. Fourth, at measurement point three (final day of assessment), one patient from the euthymic group was in a hypomanic mood state (according to YMRS score). This might also limit the results.

At the same time, the study has notable strengths: First, this study is one of the rare studies that uses empirical data in dream research with this important clinical population. Second, the study’s dropout rate was relatively low. Third, this study was controlled for: Having included and evaluated the CI emotions of healthy controls, i.e., individuals with a lifetime absence of mental disorders as evaluated by standardized diagnostic interviews, in addition to our patient group, we believe to have gathered particularly meaningful results with increased interpretability. Fourth, the CIM proved feasible: The quality criteria were very good, indicating consistent and reliable application of the CIM.

## Conclusion

Our results suggest that different emotion regulation or defense mechanisms may exist in the subgroups of bipolar disorders. Analyses of dreams and (unconscious) dream emotions may serve as helpful diagnostic and therapeutic tools and contribute to relapse prevention in the management of bipolar disorders.

## Data availability statement

The raw data supporting the conclusions of this article will be made available by the authors, without undue reservation.

## Ethics statement

This study involving humans was reviewed and approved by the ethics committee of the Charité University Medicine, Berlin (Reference number: EA4/068/13). The study was conducted in accordance with the local legislation and institutional requirements. The participants provided their written informed consent to participate in this study.

## Author contributions

GS-M: Formal analysis, Methodology, Writing – original draft. LR: Writing – review & editing, Conceptualization, Project administration, Formal analysis, Methodology. PT: Conceptualization, Methodology, Writing – review & editing, Formal analysis. WRC: Conceptualization, Formal analysis, Methodology, Project administration, Writing – review & editing. TS: Conceptualization, Methodology, Project administration, Resources, Supervision, Writing – review & editing.

## In memoriam

This paper is dedicated to Heinrich Deserno who passed away in February 2023. He came up with the initial research idea, elaborated the concept, provided crucial intellectual input, and was an important supporter of this study.

## Appendix

Medication—differences between patient groups.

**Table:**

	Euthymic	Depressive	(Hypo-)manic	Differences between patient groups^b^	
	*n* = 14	*n* = 9	*n* = 5		
Medication^a^ n (%)					
Lithium	8 (57.1%)	1 (11.1%)	1 (20.0%)	E vs. D E vs. (H)M D vs. (H)M	*p* < 0.001 *p* < 0.001 *p* = 0.53
Antiepileptics	4 (28.6%)	7 (77.8%)	5 (100.0%)	E vs. D E vs. (H)M D vs. (H)M	*p* < 0.001 *p* < 0.001 *p* < 0.001
Antipsychotics/neuroleptics	7 (50.0%)	6 (66.7%)	4 (80.0%)	E vs. D E vs. (H)M D vs. (H)M	*p* = 0.19 *p* = 0.005 *p* = 0.53
Low-potency neuroleptics	0 (0.0%)	0 (0.0%)	0 (0.0%)	–	–
Antidepressants	7 (50.0%)	7 (77.8%)	1 (20.0%)	E vs. D E vs. (H)M D vs. (H)M	*p* = 0.012 *p* = 0.005 *p* = 0.002
Benzodiazepines, Z-drugs	0 (0.0%)	0 (0.0%)	2 (40.0%)	E vs. D E vs. (H)M D vs. (H)M	*p* = 1.00 *p* < 0.001 *p* < 0.001

^a^Multiple answers were possible; in addition, some patients took more than one drug of one substance class (see Table 3). ^b^Differences (chi square tests) between patient groups concerning medication proportions; E, Euthymic; D, Depressive; (H)M, (Hypo-)manic.
